# Effects of game-based learning and flipped classroom strategies on performance and reasoning in patient safety education for surgical nursing students

**DOI:** 10.1371/journal.pone.0334545

**Published:** 2025-10-27

**Authors:** Mohadesehsadat Mehrdad, Sara Jambarsang, Hosseinali Sadeghian, Fatemeh Jabinian, Fatemeh Keshmiri

**Affiliations:** 1 Department of Nursing, Ferdows School of Medical Sciences, Birjand University of Medical Sciences, Birjand, Iran; 2 Students Research Committee, Shahid Sadoughi University of Medical Sciences, Yazd, Iran; 3 Center for Healthcare Data Modeling, Department of Biostatistics and Epidemiology, School of Public Health, Shahid Sadoughi University of Medical Sciences, Yazd, Iran; 4 Health Education and Promotion Department, School of Public Health, Shahid Sadoughi University of Medical Sciences, Yazd, Iran; 5 Operating Room Technology Department, Paramedical School, Shahid Sadoughi University of Medical Sciences, Yazd, Iran; 6 Medical Education Department, Education Development Center, Shahid Sadoughi University of Medical Sciences, Yazd, Iran; University of Pretoria, SOUTH AFRICA

## Abstract

**Introduction:**

The present study aimed to assess the effect of game-based learning (GBL) on surgical nursing students’ performance and reasoning skills in the management of patient safety incidents in surgical units compared to the flipped classroom method.

**Method:**

This quasi-experimental study was conducted at Shahid Sadoughi University of Medical Sciences in 2023–2024. One hundred surgical nursing students and surgical nurses participated in this study. In this study, a game-based learning method was implemented in the intervention group, and a flipped classroom method was used in the control group to educate the participants. The student’s reasoning skills in managing patient safety incidents were assessed by a Key Features examination. The participants’ performance was evaluated based on the WHOBARS questionnaire, which includes three sections: Sign-In, Time-Out, and Sign-out. The participants’ reasoning and performance were assessed once before the educational program and twice after the educational interventions (two weeks and two months after the educational program). Data analysis was conducted using descriptive statistics (mean and standard deviation) and Repeated Measures Analysis of Variance in SPSS software version 26.

**Results:**

The intervention group achieved significantly higher reasoning skill scores than the control group at both Post-test 1 (p < 0.001) and Post-test 2 (p < 0.001). The intervention group showed superior performance compared to the control group at Post-test 1 (p = 0.04). This difference was no longer statistically significant by Post-test 2 (p = 0.63). The effect size of the intervention on reasoning skills was large (partial η² = 0.146), while its effect on performance was moderate (partial η² = 0.03).

**Conclusion:**

This study demonstrates that game-based learning significantly enhances surgical nurses’ reasoning skills in managing patient safety incidents compared to traditional flipped learning approaches. While the intervention group showed notable short-term performance improvements at the two-week follow-up, these gains diminished over time, suggesting a need for reinforcement strategies to sustain competency.

## Introduction

Patient safety is defined as an organized framework of coordinated measures designed to ensure continuous risk mitigation, reduce the incidence of avoidable harm, lower error frequency, and lessen the severity of adverse events [1]. Among the risks to patient safety and side effects of hospitalization, the most common complications are related to surgical procedures. Retained foreign objects, wrong-site surgery, surgical site infections, falls, and injuries caused by electrosurgery are among the most common patient safety risks [2]. Although surgical adverse events are usually attributed to the skills of the surgeons, the complexity of the surgical procedures, the patient’s condition, the use of high-risk drugs during surgery, and the time limit for rapid surgical procedures in emergencies may increase patient safety risks [2]. Furthermore, the complex structure of surgical team members, management, teamwork, and organizational safety culture are among the reasons for patient safety risks in surgical departments [3].

Effective patient safety education for students across various medical disciplines has been identified as a critical debated topic. As a multifaceted concept, patient safety necessitates the development of students’ knowledge, attitudes, and skills. Furthermore, cultivating higher-order cognitive abilities is essential to enhance the speed and accuracy of decision-making in situations involving patient safety risks. Park et al. (2025), in a comprehensive meta-analysis, demonstrated that structured longitudinal educational interventions incorporating interactive methods significantly enhance nursing students’ cognitive knowledge, professional attitudes, and clinical competencies [4]. Patient safety education should include the following key components: [1] high-fidelity clinical simulations, [2] active participation in identifying and mitigating errors, and [3] training in critical thinking to improve decision-making. Further, reinforcing knowledge over time and engaging in reflective practice is crucial for sustaining learning outcomes. Therefore, it is important to integrate these elements thoughtfully into the curriculum. [5–7].

Kim et al. (2025) demonstrated that simulation-based learning and serious games effectively facilitate the application of theoretical knowledge into practical competencies within patient safety education. These active learning methods not only foster experiential learning but also significantly enhance learner engagement and long-term retention of critical safety protocols. The findings suggest that optimal educational outcomes may be achieved through blended learning approaches that strategically integrate multiple evidence-based instructional modalities to strengthen patient safety competencies in clinical practice [8]. Ferreira e al. (2025) systematically evaluated instructional methods for patient safety education, concluding that game-based learning and flipped classrooms improved learner engagement and knowledge translation in clinical safety settings. These approaches bridge theoretical and practical domains more effectively than traditional pedagogies [6]. Chang et al. used the flipped classroom method and gamification in nursing students’ patient safety education [9]. Hwang’s study that used a contextual digital game-based flipped learning approach suggested that gamification may offer broader educational benefits than standard flipped learning methods in nursing skill development [10].

### Game-based learning (GBL) in patient safety education

Luiz (2022) references the *Patient Safety Curriculum Guide* (WHO, 2011) [11] in identifying health technologies—such as interactive tutorials, online modules, competency-based training, video-assisted learning, and digital games—as effective tools for reinforcing clinical safety practices [12]. GBL is among the simulation and interactive opportunities provided for teaching important and sensitive topics in nursing education [13]. GBL is a balanced combination of challenge and learning that uses competitive elements, feedback mechanisms, and fun to engage learners in learning actively [14]. Game-based learning creates a risk-free simulation for learners to develop clinical competencies and master safety protocols, allowing repeated practice and knowledge application without endangering actual patients [15].

In the design of educational games, it is essential to observe principles such as realism, creating an entertainment atmosphere, competition, discovery, feedback, and scaffolding approach [15]. In a systematic review of game-based medical education in surgery, Graafland et al. [15] and colleagues recommended blended and interactive learning using games to teach technical and non-technical surgical skills. Ozdemir et al indicated the GBL applied in undergraduate nursing education to teach acute care surgery and surgical critical care, essential life support, triage, and decision-making processes in the surgical unit [16]. The benefits of GBL on learners’ learning include increased collaborative awareness, improved cognitive and problem-solving skills, reasoning skills, clinical decision-making, and situational awareness [14,15]. Anugrahsari’s review study (2022) [17] identified that game-based learning modalities, including educational video games, can serve as effective pedagogical tools to mitigate cognitive overload during complex patient safety instruction. This finding aligns with Dankbaar’s (2017) study, which found equivalent knowledge acquisition outcomes between video-enhanced game formats and conventional text-based e-modules. While serious games exhibit strong potential for sustaining learner attention and encouraging prolonged engagement, their effectiveness in enhancing patient safety performance remains inconsistent across studies [18]. Tan et al. (2017) developed and validated a serious game intervention in Singapore that improved nursing students’ knowledge, confidence, and clinical performance regarding safe blood transfusion protocols [19]. Game-based learning is increasingly becoming a complementary method to simulation, but contradictory results have been reported about their effectiveness. In various review studies done in 2022 and 2023, it is recommended that more studies be conducted on the efficacy of this method on learning outcomes in nursing education [13,20].

### Flipped classroom in patient safety education

The flipped classroom is a pedagogical approach in patient safety education that reverses traditional learning by having students independently study foundational knowledge (e.g., safety protocols, error prevention strategies) before class, while in-class time is dedicated to active learning through case analyses, team discussions, and problem-solving exercises. The main features of this method include pre-class resources (e.g., videos, readings), instructor-facilitated application sessions, and collaborative learning where students engage in real-world scenarios. A major benefit of the flipped classroom in patient safety education is that it enhances critical thinking and decision-making skills by allowing learners to apply theoretical knowledge in simulated risk situations. Moreover, it promotes teamwork and communication—essential competencies in patient safety—by encouraging peer interaction and collective error analysis. The flipped classroom method ensures that future practitioners are not only knowledgeable but also adept at identifying, analyzing, and mitigating safety risks in clinical settings. Its structured flexible design makes it a highly effective strategy for fostering a safety culture and continuous improvement in medical training [21–23]. Rezende et al. (2025), in a systematic review, demonstrated that the flipped classroom method coupled with interactive learning environments may enhance educational outcomes in patient safety instruction [24]. Kim et al. (2019) implemented a flipped classroom method in a patient safety course for undergraduate nursing students. The teaching methods included online learning and quizzes, case studies, small and large discussions, incident report tasks, and group projects including the development of strategies for patient safety. The flipped classroom approach significantly improved nursing students’ patient safety competencies, demonstrating positive outcomes in attitudes, clinical skills, and knowledge retention [25].

The study reveals a notable gap in formal patient safety education within the surgical nursing curriculum. This deficit underscores the critical need for rigorous interventional research to evaluate the comparative effectiveness of diverse pedagogical approaches in enhancing learner competencies, thereby informing evidence-based curricular development in this specialty domain.

To develop advanced cognitive skills for managing patient safety incidents and improve performance in patient safety education, consistent exposure to diverse clinical challenges and structured problem-solving practice is essential for effective preparedness and rapid response during risk events. To compare the effectiveness of different pedagogical strategies in patient safety education, this study examines two distinct instructional approaches: board game-based learning and the flipped classroom method. Board game-based learning was selected due to its demonstrated efficacy in fostering engagement, teamwork, and clinical decision-making through interactive, scenario-based challenges—key competencies in patient safety training. The flipped classroom approach was chosen for its systematic reinforcement of theoretical knowledge while optimizing in-class time for higher-order application of safety protocols. By juxtaposing these two methodologies—one emphasizing experiential, dynamic learning and the other structured, theory-driven instruction—this study provides a comprehensive evaluation of how different pedagogical strategies influence both learning processes and outcomes in patient safety education.

The ultimate objective of patient safety education is to enhance students’ performance and reasoning in real-life clinical environments. While prior research has predominantly assessed learning outcomes in terms of knowledge acquisition and learner engagement, there remains a need for further investigation into how educational interventions influence participants’ practical performance and clinical reasoning in patient safety education [5,17]. This study addresses this gap, offering critical insights for educators seeking to optimize training methods in this essential domain. The current study aimed to assess the effect of game-based learning on the reasoning skill in managing patient safety incidents and the performance of surgical nursing students related to patient safety compared to the flipped classroom method.

## Methods

**Fig 1:**
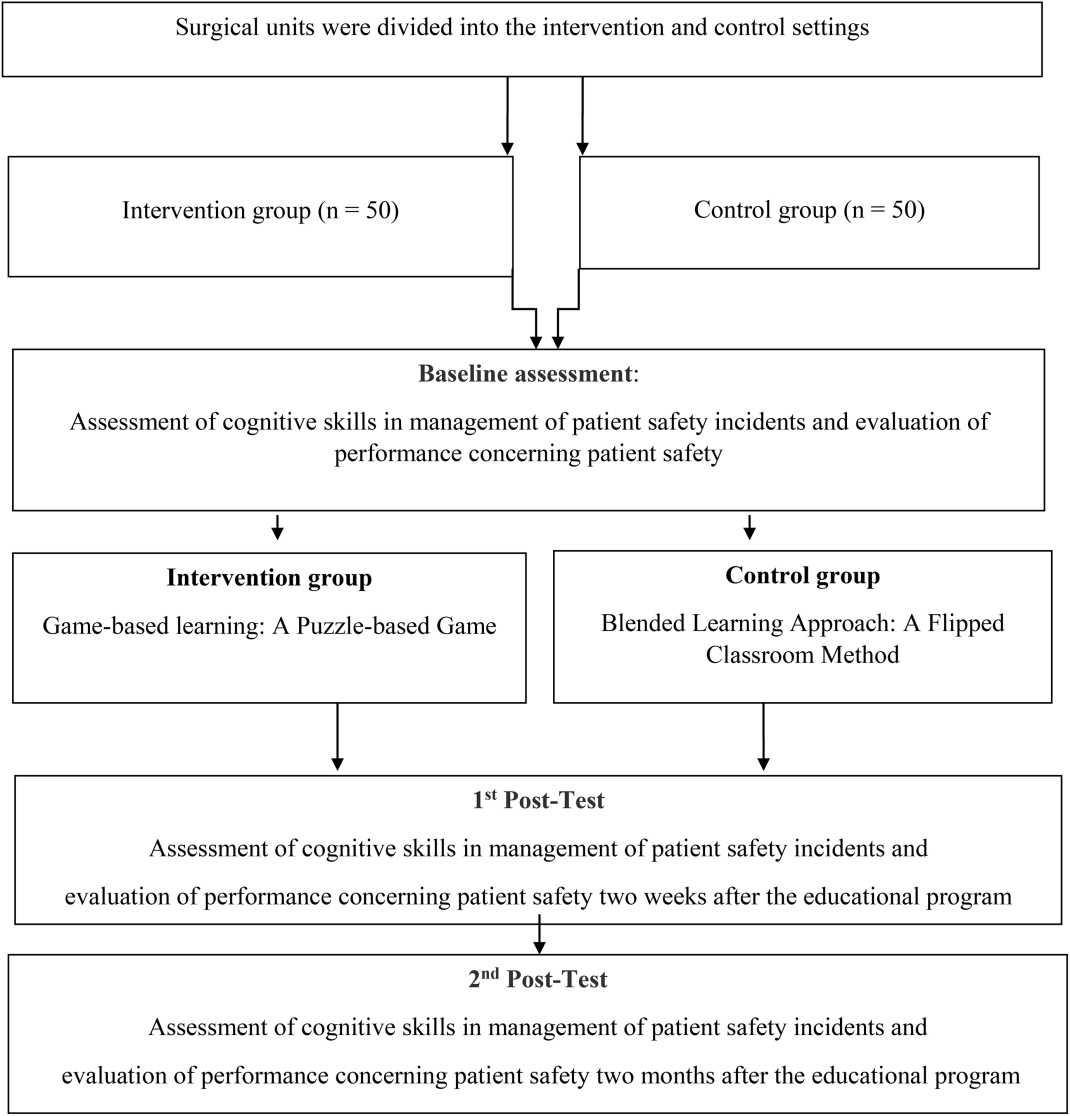
Flow Chart of the Study Steps.

**Study design.** The quasi-experimental study was conducted at Shahid Sadoughi University of Medical Sciences (SSU) on 6 July 2023 and 28 Jan 2024. (Fig 1). This study is part of a larger project on patient safety in the surgical nursing department of this university.

The study hypothesizes that there are statistically significant differences between game-based learning and flipped classroom approaches in reasoning for patient safety risk management and performance of patient safety.

### Participants

**Learners:** Eligible participants included surgical nursing students who had undertaken six-month surgical unit internships and practicing surgical nurses with six or more months of clinical experience in surgical team settings. We excluded individuals with prior error management/safety training, those declining participation, personnel with <6 months’ surgical experience, and staff transferring units mid-study.

The sample size was determined through an a priori power analysis based on three key parameters from our pilot study: α = 0.05 (two-tailed), power (1-β) = 0.80, and effect size (d) = 0.5. Initial calculations indicated a minimum requirement of 31 participants per group. To ensure robustness, we incorporated three additional safeguards: [1] a 30% buffer for potential attrition (n = 40/group), [2] compensation for design effects from cluster randomization, and [3] adequate power for planned analyses. These considerations yielded a final sample of 100 participants (50 per group), which exceeded requirements as actual attrition remained below projected levels.

The study employed a cluster-randomized design involving multiple surgical units. Both surgical nursing students and surgical nurses were assigned to either intervention or control groups based on their affiliated surgical unit’s randomization. All participants working within a particular unit were automatically allocated to the same study arm (intervention or control) as their respective unit, ensuring complete adherence to the cluster randomization protocol.

**Facilitator:** Four educators participated as a facilitator team with a mean age of 38 years (SD = 6) and a mean work experience of 6 years (SD = 2). Three of the educators were experienced surgical nurses (75%), while one was an expert in medical education (25%).

### Educational intervention

In this study, educational interventions were implemented using two game-based learning methods in the intervention group and the Flipped classroom method in the control group.

### Game-based learning in the intervention group

#### Puzzle-based game design.

In the present study, a puzzle-based game was designed by observing the four elements of game-based pedagogy. Cognitive engagement encourages students to think analytically, apply their knowledge, and adapt to evolving situations in the game by stimulating cognitive processes through presenting challenging patient safety situations in the surgical unit. Behavioral engagement encourages active participation and problem-solving in the game and shapes students’ behavior in interacting with game challenges. In the game’s design, students were involved with the riddles of each puzzle to develop critical thinking and decision-making skills. Social engagement happens through peer interaction and participation among group members in playing and creating competition. The puzzle board game was designed to be played in groups so that students in each group could exchange ideas and viewpoints with their team members. They had the opportunity to learn from each other and solve educational puzzles together. In the affective element, the puzzle game uses emotional and motivational factors to make learning enjoyable and meaningful. Puzzles create curiosity and excitement among students by providing riddles. Receiving points and creating competition between groups cause a positive learning environment that motivates participants to obtain more marks.

The educational puzzles were meticulously designed to address eight common patient safety risks. These errors encompassed areas such as safe patient admission before surgery, the safe surgery checklist in the critical stages of Sign-In, Time-Out, and Sign-Out, unintended Retained Surgical Items (RSI) prevention, safety measures for medical gas cylinders, the safe preparation and sending of pathology samples, and the prevention of pressure injuries.

### Game-based learning process

#### Briefing phase.

The briefing session was implemented to familiarize the students with the teaching method and present the educational content before the training program. The educational materials including the patient safety scripts and info-graphics of common incidents were explained and sent to the participants through an electronic link one week before the training program. The participants were asked to read the training materials before attending the face-to-face sessions.

Each game included a scenario that explained an error related to the surgical units, a matrix game, and instructions detailing the gameplay to solve the problem in a game-based learning process.

According to the instructions, the puzzle game process started. First, the puzzle shuffled cards were classified into ISBAR process ((Identification, Situation, Background, Analysis, and Recommendation) as a structured dialogue tool during handover), World Health Organization (WHO) safe surgery checklist, safety measures in unintended RSI, recognition of medical gases in cylinders, sampling, collecting and sending pathology samples to the laboratory, and safety of pressure injuries related to patient positioning.

After classifying the puzzle pieces individually, the learners in the small groups were asked to complete the matrix according to each of the puzzle steps. Based on the steps described in the instructions, the learners discussed and talked about choosing the components of the educational puzzle. They had the opportunity to consult and review educational materials during the game. During this process, the facilitator was leading students toward teamwork and engagement. To optimize systematic participation, all small-group members rotated through predefined, structured roles during game-based learning sessions (e.g., scenario reader, solution proposer, checklist verifier). Trained facilitators actively monitored group dynamics, employing targeted prompting strategies to ensure equitable contribution from all participants, with particular attention to less vocal learners.

In the second step, students engaged in peer evaluation, assessing the puzzles created by other groups. Each group had the opportunity to refine their puzzles based on the feedback received. Scores for each group were determined based on the completed components of each puzzle. After completing the puzzles, the facilitator, who is an experienced healthcare professional, provided constructive feedback to the students, which allowed them to enhance their work. The feedback and summary from the facilitator aimed to offer comprehensive guidance and closure to the process.

### Flipped classroom in control group

The Flipped classroom method encompasses two distinct parts: 1) learning at-home material and 2) engaging in exploratory and peer-collaborative activities during in-class sessions [26]. In the first step, students completed direct instruction by studying educational material on patient safety incidents, including educational scenarios, patient safety risk scripts, and info-graphics, before the face-to-face training sessions. In the second step, the concept of “patient safety risks in the surgical units” was discussed in the classroom session. This was followed by analyses of examples and cases related to patient safety risks, employing a root cause analysis technique.

#### Study duration and frequency.

The training program was implemented monthly for four months.

### Evaluation

#### Learning assessment.

***Reasoning Skills Assessment:*** Participants’ high-level cognitive skills in managing patient safety incidents were evaluated using a longitudinal assessment design. A Key Features Examination in a paper-based format was used to assess the reasoning skills of participants (refer to the Study Measures section for detailed information on the instrument’s characteristics). Baseline measurements were collected before the educational intervention. Two post-intervention assessments were conducted for knowledge retention at two-week and two-month intervals.

***Performance Assessment*:** Participant performance was assessed through structured observations by a trained evaluator—an experienced surgical nurse with six years of clinical practice. Before data collection, the evaluator underwent comprehensive training to ensure standardized application of the assessment protocol. This training included detailed instruction on the proper use of the evaluation tool, adherence to structured observation methods, and the requirement for conducting a minimum of three independent observations per participant. To minimize bias, the evaluator remained blinded to participant group allocation throughout all assessments. The training protocol incorporated competency verification through practice observations and inter-rater reliability checks to ensure consistent and objective scoring. Performance was evaluated across critical phases of surgical care—including Sign-In, Time-Out, and Sign-Out— during patient safety–specific scenarios, using the validated WHOBARS checklist. Each participant was assessed at three-time points: baseline (pre-intervention), post-intervention (two weeks), and follow-up (two months), with final performance scores derived from the mean of three observational assessments per time point to enhance reliability. (see Study Measures section).

### Study tools

In this study, the Key Feature (KF) examination, WHOBARS questionnaire, and participant demographic information worksheets were completed. Demographic information included age, sex, and position (worker vs. student).

The students’ reasoning skills in managing patient safety incidents were evaluated through KF Examination (15 Questions). Two surgical nursing specialists compiled the questions. The validity of the KF examination was reviewed and confirmed by 15 experts. Each KF question consisted of one scenario and 12 options. Students were asked to choose three options in each question. The scoring range in the test was between 0–45.

The evaluation of participants’ performance was based on the WHOBARS questionnaire, which was first designed by Devcich et al. [27] as a behavioral rating scale at three strategic surgery times in 2016. This questionnaire includes three sections: Sign-In (before the patient’s anesthesia induction), Time-Out (before cutting the skin), and Sign-Out (before the patient leaves the operating room), and each section contains five items. The psychometrics of the mentioned instrument were approved by the authors (Cronbach’s Alpha 0.86, ICC = 0.81) [28]. The learners’ performance was scored from 1 (poor) to 7 (excellent), and the minimum and maximum scores were 15 and 105, respectively.

### Data analysis

The data were analyzed using descriptive statistics (mean and standard deviation) and Repeated Measures Analysis of Variance in SPSS software version 26. Partial Eta Squared was calculated to determine the effect size of the educational intervention. The level of educational effect was categorized as low (0.01), moderate (0.06), or high (0.1) [29]. The significant level was considered 0.05.

### Ethics consideration

This study is part of a larger study on patient safety in surgical nursing. This study was approved by the Ethics Committee of Shahid Sadoughi University of Medical Sciences, Yazd, Iran. (ID: IR.SSU.SPH.REC.1402.034). The work was conducted following the Declaration of Helsinki. All participants were provided with information on the study and gave consent. The written consent forms were obtained from all participants. During manuscript preparation, an AI-based language model was utilized for proofreading and structural refinement while maintaining academic rigor.

## Results

In this study, 100 students and workers in surgical nursing participated in the intervention group (n = 50) and the control group (n = 50). Table 1 shows the demographic information of the participants.

**Table 1:** The demographic characteristic of participants.

Demographic Information	Category	Intervention group N (%)	Control group N (%)	P-Value
**Gender**	Female	31 (62)	27 (54)	0.418
	Male	19 (38)	23 (46)	
**Age**	21-30	34 (68)	34 (68)	0.145
	31-40	9 (18)	14 (28)	
	41-50	7 (14)	2 (4)	
**Position**	Worker	30 (60)	30 (60)	>0.999
	Student	20 (40)	20 (40)	

The results demonstrated a significant improvement in management reasoning skills among the intervention group compared to the control group over time, with a high educational effect (Partial η² = 0.146). After adjusting for position and age, no significant difference in reasoning skill scores was observed between the two groups at baseline (p = 0.12). The intervention group achieved significantly higher reasoning skill scores than the control group at both Post-test 1 (p < 0.001) and Post-test 2 (p < 0.001). After adjusting for pre-test scores, the time-intervention interaction was significant (p < 0.001), with the intervention group exhibiting higher reasoning skill scores (p < 0.001). Both groups showed significant within-group improvements (p < 0.001), though the intervention group’s gains were consistently greater (Table 2).

**Table 2:** The score of reasoning skills in the management of patient safety incidents of participants in control and intervention groups over time.

Time*	Intervention Group	Control group	Comparison of groups	Partial eta squared	Time-group interaction
	Mean ± SD	Mean ± SD	p-value		P-value^§^
**Pre-test**	19.68 ± 3.461	20.68 ± 3.047	0.128	0.146	<0.001^§^
**1**^**st**^ Post-Test**	27.82 ± 3.916	23.58 ± 2.800	<0.001^§^		
**2**^**nd**^ Post-Test***	30.88 ± 3.520	24.80 ± 3.307	<0.001^§^		
**Comparison of times** **P-value**^**§**^	<0.001^§^	<0.001^§^			

* After adjusting for age and position.

**Two weeks after the intervention.

*** Two months after the intervention.

§The significant level was considered 0.05.

The results indicated a significant improvement in performance scores in the intervention group compared to the control group over time (p < 0.001), with adjustments for pre-test scores. The time-intervention interaction was significant (p < 0.001). After controlling for position and age, the baseline performance scores did not differ significantly between groups (p = 0.67), the intervention group showed superior performance compared to the control group at Post-test 1 (p = 0.04). This difference, however, was no longer statistically significant by Post-test 2 (p = 0.63). Both groups showed significant within-group changes over time (intervention: p = 0.001; control: p < 0.001), though the intervention group consistently scored higher. The intervention had a moderate educational effect (Partial η² = 0.034) (Table 3).

**Table 3:** The performance score of the participants in the two intervention and control groups over time.

Time*	Intervention Group	Control group	Comparison of groups	Partial eta squared	Time-group interaction
	Mean ± SD	Mean ± SD	p-value		P-value^§^
**Pre-test**	50.88 ± 2.608	50.66 ± 2.631	0.675	0.034	0.073^§^
**1**^**st**^ Post-Test**	53.22 ± 3.222	52.04 ± 2.523	0.044^§^		
**2**^**nd**^ Post-Test***	51.88 ± 2.616	51.64 ± 2.439	0.636		
**Comparison of times** **P-value**^**§**^	0.001^§^	<0.001^§^			

After adjusting for age and position.

**Two weeks after the intervention.

*** Two months after the intervention.

§The significant level was considered 0.05.

## Discussion

The present study showed that game-based learning significantly improved the participants’ reasoning skills to solve patient safety incidents and their performance compared to the control group. The game-based learning was reported to have a large educational effect on the reasoning skill to manage patient safety and a moderate effect on participants’ performance related to patient safety.

The present results showed that the reasoning skill scores of the manage patient safety risk were significantly improved in the intervention group compared to the control group over time. The participants in the intervention group learning through the puzzle-based method were able to make learning better and more retention compared to the control group. Game-based learning immerses students in interactive, scenario-based challenges that simulate real clinical risks, requiring collaborative problem-solving and immediate decision-making. This method enhances engagement, teamwork, and critical thinking, as learners actively apply safety protocols in a dynamic, game-like setting. The observed longitudinal improvement in reasoning scores may be attributable to the iterative problem-solving processes inherent in GBL, which provide repeated cycles of experiential learning and immediate feedback. The results showed that the reasoning scores of the control group participants had improved compared to before the intervention, but over time, the intervention group’s scores were reported to be higher than the control group. The participants in the control group also read the scripts of safety risks before the class, and they engaged with patient safety challenges and solved them through examples and cases presented in the class. Consistent with the present study, Frey acknowledged that learners’ participation in games related to high-risk environments, such as the surgical unit and emergency department, promotes the development of non-technical skills, such as reasoning and decision-making of learners [30]. The study by Surapaneni et al. utilized “*CARBGAM*” *(Card & Board Games in Medical Education)* to teach complex topics, finding that the intervention group showed significantly higher cognitive scores compared to the traditional lecture-based control group [31]. This aligns with the present findings, suggesting that problem-solving, engagement, and competitive elements in GBL enhance learning outcomes. Chang’s investigation of the flipped classroom method and gamification in nursing students’ patient safety education revealed a significant improvement in collective efficacy and patient safety competency over time [9]. Hwang’s study found that a contextual digital game-based flipped learning approach was more effective than traditional flipped learning for teaching intravenous injection skills. Students using the approach demonstrated greater comprehension, higher academic performance, and improved motivation compared to those in the conventional flipped classroom. Moreover, they exhibited stronger engagement, more positive learning attitudes, and enhanced critical thinking tendencies [10]. Likewise, in our game, the students in the intervention group encountered different scenarios during the puzzle-based learning, where they experienced difficult situations to manage. Hence, the opportunity for interaction and collaborative learning in small groups can effectively develop reasoning skills to manage patient safety risks under challenging situations among students. Zamani’s (2021) study demonstrated that blended learning (combining word puzzles with lectures) significantly improved knowledge retention and learning scores in the intervention group compared to traditional lectures, both immediately post-training and after one month [32]. These findings align with the present study, reinforcing that game-based learning enhances long-term retention through challenge-based engagement, repeated decision-making, and immediate feedback in small-group settings. The improvement in second post-test scores further supports the sustained benefits of active, game-based learning strategies.

The results indicated that the participants’ exposure to challenging scenarios and trial and error to solve various puzzle problems in small groups positively affected the performance of participants regarding patient safety. The present showed the GBL had a positive and significant effect on the participant’s performance in the intervention group compared to the control group after two weeks. They could practice and receive feedback on the GBL, which may affect the results. The findings of Gudadappanavar and Coelho’s studies corroborate the positive impact of puzzle games on participants’ performance and align with the present results [33,34]. XU et al. [35] acknowledged that serious games can raise cooperative awareness through students’ exposure to different game settings, providing active learning opportunities to solve clinical problems and improve clinical performance and decision-making skills, which aligns with the present results. Akbari et al. (2022) [36] conducted a study utilizing a virtual game to teach novice surgical technology students about the tools and equipment used in the surgical unit. Their findings revealed that the intervention group, who played the virtual game, exhibited higher scores, made fewer errors, and earned more rewards compared to the control group, who received traditional lectures on surgical table setup. The opportunity to practice, receive feedback on their decisions, and engage in group discussions during the game process may contribute to positive changes in participants’ performance scores when applied in real-world settings. The results of the review study by Min et al. [37] revealed that educational games effectively improve students’ performance. Creating the opportunity to engage with problems, experience different situations, reach various solutions, and receive feedback can affect the improvement of participants’ performance in the game. The study found that while the flipped classroom method in the control group effectively facilitated foundational knowledge acquisition through pre-class learning and in-class application (discussions, case analyses, and exercises), its performance outcomes remained lower than those of the intervention group. This discrepancy may be attributed to inadequate pre-class preparation, which likely diminished the effectiveness of in-class activities and compromised long-term knowledge retention and practical competency development.

This study revealed two critical findings regarding patient safety training outcomes: (1) the educational intervention showed moderate effect in improving performance measures, and (2) both teaching approaches demonstrated notable performance deterioration over time, with two-month retention scores significantly lower than two-week post-intervention results. The results demonstrate that both game-based learning and flipped classroom approaches effectively enhance cognitive skills over time. However, achieving lasting behavioral change requires complementary methods that strengthen experiential learning components, and systematically address environmental and organizational factors influencing behavior sustainability. Since behavior change is a complex issue, in addition to education, it requires changing various factors in the environmental process, the development of systems, and supportive mechanisms.

This study recommends the implementation of GBL supplemented with reinforcement strategies to enhance patient safety training among surgical nurses. Furthermore, it proposes future longitudinal research to evaluate the efficacy of hybrid instructional models integrating GBL with flipped classroom approaches. Additional investigations should examine both the immediate effects on performance outcomes and the long-term retention of competencies, which may address existing gaps in learner engagement and the practical application of skills in clinical settings.

## Limitations of the study

This study has several limitations that should be acknowledged. First, the quasi-experimental design, without randomization or blinding, may introduce selection bias and limit causal interpretations, despite statistical adjustments for baseline differences. Second, the relatively small sample size (n = 100) from a single institution restricts the generalizability of findings to other settings or populations. Third, while we standardized protocols, variations in intervention fidelity (e.g., inconsistent pre-class preparation in the flipped group or uneven team participation in puzzle sessions) could have influenced outcomes. Fourth, reliance on a single trained assessor for performance evaluations, though intended to ensure consistency, may have introduced measurement bias; future studies would benefit from multiple blinded raters. Finally, the 2-month follow-up period provides only preliminary evidence of knowledge retention; a longer-term assessment is needed to evaluate sustained competency gains. These limitations highlight opportunities to strengthen future research through randomized controlled designs, multi-site recruitment, and more robust evaluation methods.

## Conclusion

The present study demonstrated a substantial educational effect of the game-based learning method on participants’ reasoning skills in managing patient safety incidents compared to the flipped learning approach (large effect size). Moreover, a moderate intervention effect was observed on performance scores. This study demonstrates that game-based learning significantly enhances surgical nurses’ reasoning skills in managing patient safety incidents compared to traditional flipped learning approaches. While the intervention group showed notable short-term performance improvements at the two-week follow-up, these gains diminished over time, suggesting a need for reinforcement strategies to sustain competency.

Given the critical role of patient safety in surgical settings, these findings support the integration of GBL into nursing education programs—both as formal training modules and informal learning tools. To maximize long-term impact, future implementations should incorporate periodic refresher sessions and adaptive difficulty levels tailored to learners’ experience. Further research should explore optimal reinforcement intervals, hybrid GBL-simulation models, and longitudinal competency retention in clinical practice.
